# The Effectiveness of COVID-19 Vaccines During the Pre-Omicron and Omicron Periods: A Retrospective Test-Negative Case–Control Study

**DOI:** 10.3390/vaccines12111245

**Published:** 2024-10-31

**Authors:** Romeo Brambilla, Renata Gili, Federica Vigna Taglianti, Jacopo Lenzi, Matteo Riccò, Roberto Burioni, Mariaelisabetta Scarvaglieri, Rachele Rocco, Vittorina Buttafuoco, Rosa Maria Teresa Antonia Cristaudo, Davide Gori

**Affiliations:** 1 Local Health Unit of Torino, Department of Prevention, Via Silvio Pellico 19, 10125 Turin, Italy; 2 Department of Microbiology and Virology, Università Vita Salute San Raffaele Medical School, Via Olgettina 58, 20132 Milan, Italy; 3 Department of Translational Medicine, University of Eastern Piedmont, Via Solaroli 17, 28100 Novara, Italy; 4 Department of Biomedical and Neuromotor Sciences (DIBINEM), University of Bologna, 40126 Bologna, Italy; 5 AUSL–IRCCS di Reggio Emilia, Servizio di Prevenzione e Sicurezza Negli Ambienti di Lavoro (SPSAL), Local Health Unit of Reggio Emilia, 42122 Reggio Emilia, Italy

**Keywords:** vaccination, COVID-19, effectiveness, test-negative design, booster, bivalent vaccine

## Abstract

Background: The aim of this study was to estimate the effectiveness of original and bivalent COVID-19 vaccines in reducing COVID-19-associated hospitalizations among the adult population of Turin, Italy. Methods: We conducted a retrospective, test-negative, case–control study of 5768 adults aged ≥50 years who had symptoms that were consistent with COVID-19-like illness and were admitted to the hospitals of the Turin Health Unit network from 1 January 2021 to 31 January 2023. We evaluated the effectiveness of the vaccines that at the time of the study were authorized in the European Union (original/bivalent BNT162b2; original mRNA-1273; ChAdOx1-S; Ad26.COV2.S) by comparing the odds of a positive test for SARS-CoV-2 in vaccinated patients with the odds of a positive test in unvaccinated patients. The association between vaccination status, hospitalization, ICU admission and positive SARS-CoV-2 test was estimated by building multivariate adjusted logistic regression models. Results: During the predominance of the pre-Omicron variants, the vaccine effectiveness of two and three doses received in the last 120 days against COVID-19-associated hospitalizations was 93.6% (95% CI: 90.1 to 95.9) and 97.1% (95% CI: 90.8 to 99.1), respectively. During the predominance of the Omicron variant, the vaccine effectiveness of two and three doses was 26.6% (95% CI: −0.6 to 46.5) and 75.2% (95% CI: 68.1 to 80.7), respectively, and it rose to 88% (95% CI: 78.2 to 93.3) for four or five doses of the bivalent vaccine. Conclusions: Our study confirms that the COVID-19 vaccines protect adult patients from hospitalizations, including the subgroup ≥80 years, also during the period of the Omicron variant’s predominance.

## 1. Introduction

The Omicron variant (B.1.1.529) of the Severe Acute Respiratory Syndrome CoronaVirus 2 (SARS-CoV-2) led to a surge in new cases of an unprecedented magnitude worldwide. Later, further Omicron sublineages emerged, creating a complex panorama of variants known as the “variant swarm” [1].

Differently from what happened with the Alpha and Delta variants, with the appearance of Omicron, a certain reduction in vaccine effectiveness was observed [2]. This reduction was due to both the waning of immunity [3,4,5] and the capability of Omicron to evade the host’s immune response [6,7]. The decline in immunity, in line with what has been observed for other vaccines [8,9,10], was higher in people who were older than 55 years and in individuals with comorbidities [3]. Fortunately, the vaccine protection against severe COVID-19 appeared to remain high [11,12,13]. In addition, Omicron caused a less severe disease than the precedent circulating variants [2,14,15].

Despite the remarkable vaccine effectiveness against severe disease, the lower pathogenicity of Omicron and the high vaccination coverage among the Italian population, elderly and frail people—even if vaccinated—maintained a higher risk of severe outcomes than young people [16]. Therefore, the Italian Ministry of Health, similar to other countries, has run a campaign promoting a fourth dose of the vaccine from April 2022 [17]. Moreover, in September 2022, the European Medicines Agency (EMA) authorized two updated bivalent mRNA vaccines and recommended their use as booster doses [18]. Given the many vaccines available, many people received combinations of different types of vaccines. However, studies showed that these “mix-and-match” regimens offer good protection against the disease [19]. In such a dynamic situation and for the benefit of future epidemics, the evaluation of the duration of vaccine protection against severe outcomes and the right timing for boosters should be encouraged [20].

Our study aims to estimate the effectiveness of original and bivalent COVID-19 vaccines in reducing COVID-19-associated hospitalizations and admissions to intensive care units (ICUs), by age and time since administration, in the adult population of Turin, north-western Italy, and to assess differences in vaccine effectiveness under the predominance of the pre-Omicron and Omicron variants.

## 2. Materials and Methods

### 2.1. Setting, Study Population and Data

This study was conducted in Turin, a city of around 900,000 inhabitants in north-western Italy. The study population included adults aged ≥50 years residing in Turin with symptoms that were consistent with COVID-19-like illness who were admitted to hospitals in the city’s Health Unit network between 1 January 2021 and 31 January 2023.

A COVID-19-like illness was defined by a clinical diagnosis of acute respiratory illness (e.g., respiratory failure or pneumonia) or the presence of at least one sign or symptom (e.g., cough, fever, dyspnea, vomiting or diarrhea) related to COVID-19. These diagnoses and symptoms were identified in the registry of discharge forms using the codes of the ninth revision of the International Classification of Diseases (ICD-9), similar to the study by Thompson [21,22]. Hospital readmissions within 30 days were pooled and analyzed as single hospitalization events.

To determine the ability of the vaccine to protect against SARS-CoV-2 infection, patient records were anonymized through a pseudo-anonymization code that was used for data linkage with the vaccination registry and regional database of COVID-19 tests. In order to reduce the influence of protection due to previous infections, patients who had a previous SARS-CoV-2 infection documented in their medical records before the hospital admission were considered not eligible. Moreover, patients must have had the opportunity to have been vaccinated at the date of hospital admission. In this regard, a subject was considered potentially eligible only if the hospital admission occurred at least 21 days after the local availability of the first COVID-19 vaccine dose for his/her age group. Finally, only subjects with a SARS-CoV-2 molecular or antigen test executed within the 14 days before through to 72 h after the hospital admission were eligible for this study.

The following information was available and extracted for the analysis: sociodemographic characteristics (age and gender), vaccination status (date of vaccination and number of doses), COVID-19 tests (date and result) and hospitalization data (admission date, discharge date, ward, primary and secondary diagnoses, comorbidities and Charlson Comorbidity Index—CCI). Every patient was tested for SARS-CoV-2 infection using a molecular or antigen test at the moment of the admission to the hospital. There were no missing data for the sociodemographic characteristics and information on hospitalization. Subjects who were not registered as vaccinated in the regional vaccination register were considered unvaccinated.

Critical illnesses requiring mechanical and assisted ventilation can be treated in various settings, not only in the ICU. Therefore, both actual ICU admissions and the use of mechanical ventilation or Continuous Positive Airway Pressure (CPAP) during hospitalization were considered ICU admissions. In the first case, the ICU was defined according to the department of hospitalization; in the second case, the use of mechanical ventilation or CPAP was derived from the ICD-9 codes of the intervention as registered in the Hospital Discharge Form.

Based on the data from the Italian National Institute of Health [23], hospitalizations occurring from 1 January 2021 to 15 December 2021 were attributed to the pre-Omicron predominance period, whilst those occurring from 16 December 2021 to 31 January 2023 were attributed to Omicron. Indeed, at the beginning of January 2022, the Omicron variant was at 81% of prevalence [23], meaning that it had already been the prevalent variant in Italy for at least a few days. Considering, then, that from the beginning of the COVID-19 pandemic, Italian National Institute of Health data were characterized by a notification delay of approximately 10–15 days [24], the date of 15 December 2021 was chosen as the date indicating the change in variant predominance.

### 2.2. Study Design

Retrospective test-negative case–control design.

### 2.3. Definition of Cases and Controls

A case was defined as a hospitalized patient with at least one ICD-9 code that was consistent with a COVID-19-like illness and at least one positive SARS-CoV-2 result on tests (molecular or antigen) performed between 14 days before and 72 h after hospital admission.

A control was defined as a hospitalized patient with at least one ICD-9 code that was consistent with a COVID-19-like illness and only negative SARS-CoV-2 results on tests (molecular or antigen) performed between 14 days before and 72 h after the hospital admission.

### 2.4. Vaccination Status

In order to assess vaccination exposure, all the vaccines that at the time of the study were authorized in the European Union were considered: (1) BNT162b2—original and bivalent formulation; (2) mRNA-1273—original formulation; (3) ChAdOx1-S; and (4) Ad26.COV2.S. The bivalent formulations were authorized by the Italian Drug Agency in September 2022 and have been used starting from this month.

Vaccination status was documented using the regional vaccination registry and categorized according to the number of doses received and the number of days from the administration of the last vaccine dose. Patients were considered unvaccinated if they did not receive any dose of vaccine or if they received only one dose. Patients with two doses were further divided into two categories according to the time from the administration of the second dose: from 7 to 120 days and >120 days. In case of three doses, the categories were defined according to the time from the administration of the third dose: 1–120 days or >120 days. Due to the relatively low number of people who received the fourth and the fifth dose in our sample, this class was not further categorized. For this last group, we made a further subdivision according to the type of formulation administered: original vs. bivalent.

Given the high complexity and the large number of different possible vaccine combinations used throughout the study period, vaccine subgroup effectiveness analyses could not be performed. The distribution of COVID-19 vaccines administered to the study sample, overall and by time period, is shown in Table S1.

### 2.5. Study Sample

From 1 January 2021 to 31 January 2023, a total of 10,535 hospitalizations for a COVID-19-like illness occurred in the hospitals belonging to the Turin Health Unit network among patients ≥50 years old residing in Turin. Of these, 1720 admissions occurred within the 30 days following the previous discharge and were therefore summarized and analyzed as single hospitalization events. A further 1757 hospitalizations occurred before the age-specific COVID-19 vaccine availability date and were consequently excluded. Finally, 1290 hospitalizations were patients with a documented previous COVID-19 infection. These exclusions resulted in a final sample of 5768 patients who were eligible for this study.

### 2.6. Statistical Analysis

Categorical variables were presented as frequencies and percentages, while continuous variables were described using the mean and standard deviation. Simple logistic regression analysis was employed to assess unadjusted differences in demographic and clinical characteristics between test-positive cases and test-negative controls, providing crude odds ratios (cORs).

Following the approach by Thompson [21], propensity-for-vaccination scores were first calculated for test-negative controls, and these scores were then applied to the test-positive group using an adjusted model [21,25]. Since the test-negative design can be seen as an indirect cohort approach and COVID-19 is not an uncommon outcome, all the observations were included in a sensitivity analysis to estimate the propensity-for-vaccination scores, as in Thompson’s study [21,26].

To estimate the propensity to be vaccinated, Multiple Additive Regression Trees (MARTs) with gradient boosting were used, incorporating explanatory variables such as sex, age and the Charlson Comorbidity Index (CCI) [21,27]. The following regularization parameters were applied: a maximum tree depth of 5, up to 20,000 iterations, 50% bagging and a shrinkage factor of 0.01. Given the inclusion of multiple treatment levels (no vaccination/1 dose, 2 doses [≤/>120 days], 3 doses [≤/>120 days], and 4–5 doses [original/bivalent]), a multinomial distribution was utilized for vaccination outcome predictions from the boosting models.

The primary variable of interest was the average treatment effect in the population, with each observation being weighted by the inverse probability of receiving the treatment that was actually administered (inverse probability treatment weighting [IPTW]). Separate weights were generated for each logistic vaccine effectiveness model, and weights exceeding the 90th percentile were truncated to mitigate extreme values, reducing bias and variance while preserving overlaps in propensity scores across treatment groups and maintaining statistical power [28]. Additionally, to account for potential residual confounding after the boosting process [21,27], a doubly robust estimation method was used: sex, age and CCI were centered at the unweighted sample mean and included again as covariates in the vaccine effectiveness regression models, with squared terms added to augment the model specification.

Vaccine effectiveness was derived using multivariable logistic regression analysis, which controlled for potential confounders in the relationship between vaccination status and laboratory-confirmed SARS-CoV-2 infection by including predefined covariates and weights based on the propensity-for-vaccination scores.

The vaccine effectiveness from the logistic models was calculated as (1 − OR) × 100. The odds ratio for hospitalization represented the odds of hospitalization related to SARS-CoV-2 infection among vaccinated individuals versus unvaccinated individuals. Similarly, the odds ratio for ICU admission compared the odds of ICU admission between vaccinated and unvaccinated individuals with SARS-CoV-2 infection. The vaccine effectiveness was further evaluated by age group by dividing the data into 50–79 and ≥80 years categories. The same analytic framework was applied for these stratified vaccine effectiveness estimates as used in the primary analysis.

All statistical analyses were performed using Stata 17 (StataCorp. 2021. Stata Statistical Software: Release 17. College Station, TX, USA: StataCorp LLC).

## 3. Results

### 3.1. Characteristics of the Study Sample

The sample included 5768 patients ≥50 years of age residing in Turin and hospitalized for COVID-19-like illness in the hospitals belonging to the Turin Health Unit network from 1 January 2021 to 31 January 2023. Of them, 2083 (36.1%) occurred during the pre-Omicron variants’ predominance period (Table 1), and 3685 (63.9%) occurred during the Omicron variant’s predominance period (Table 2).

During the predominance of the pre-Omicron variants, the mean age of the admitted patients was 78.4 years (±10.6); 45.9% of them were females and 54.1% were males (Table 1). The subsamples of cases and controls did not differ in terms of gender and age, whilst differences were detected for vaccination status, CCI and ICU admission rates (Table 1).

During the predominance of the Omicron variant, the mean age of the admitted patients was 78.2 years (±11.0); 48.3% of them were females and 51.7% males (Table 2). The subsamples of cases and controls did not differ in terms of gender, age and ICU admission rates, whilst differences were detected for CCI and vaccination status (Table 2).

### 3.2. Vaccine Effectiveness on Hospitalizations

**Figure 1:**
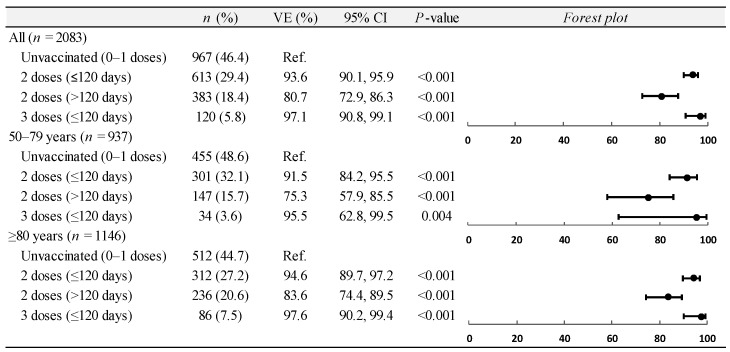
Estimates of average vaccine effectiveness (VE) against COVID-19-associated hospitalization among adults aged ≥50 years during predominance period of SARS-CoV-2 pre-Omicron variants (1 January to 15 December 2021), overall and by age group. COVID-19, coronavirus disease 2019; SARS-CoV-2, severe acute respiratory syndrome coronavirus 2; CI, confidence interval.

During the predominance of the pre-Omicron variants, the vaccine effectiveness against COVID-19-associated hospitalizations in the overall population of adults ≥50 years was 93.6% (95% CI: 90.1 to 95.9) for two doses administered within less than 120 days; 80.7% (95% CI: 72.9 to 86.3) for two doses administered within more than 120 days; and 97.1% (95% CI: 90.8 to 99.1) for three doses administered within less than 120 days (Figure 1). The effectiveness of two and three doses administered after less than 120 days was above 90% in both patients aged 50–79 years and patients aged 80 years or older (Figure 1). The estimates of vaccine effectiveness remained virtually unchanged in the sensitivity analyses (Table S2).

**Figure 2:**
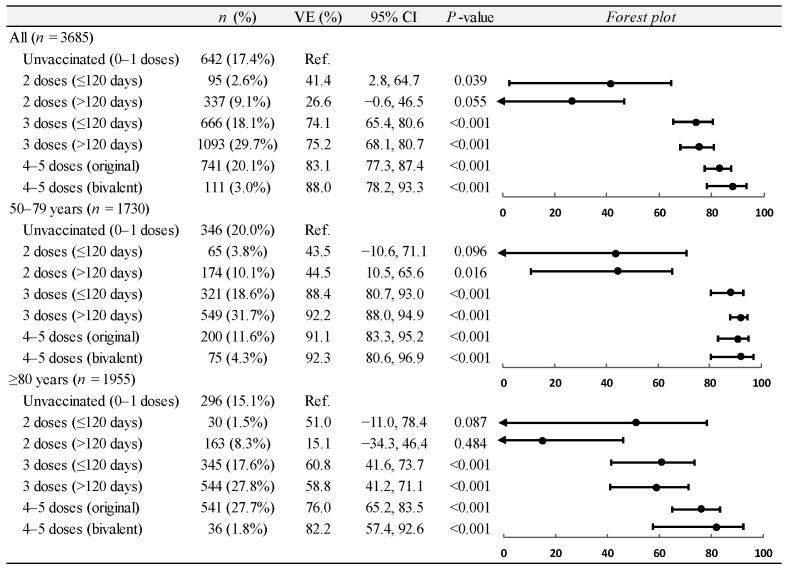
Estimates of average vaccine effectiveness (VE) against COVID-19-associated hospitalization among adults aged ≥50 years during predominance period of SARS-CoV-2 Omicron variants (16 December 2021 to 31 January 2023), overall and by age group. COVID-19, coronavirus disease 2019; SARS-CoV-2, severe acute respiratory syndrome coronavirus 2; CI, confidence interval.

During the predominance of the Omicron variant, the vaccine effectiveness against COVID-19-associated hospitalizations among adults ≥50 years was 41.4% (95% CI: 2.8 to 64.7) for two doses administered within less than 120 days; 26.6% (95% CI: −0.6 to 46.5) for two doses administered within more than 120 days; 74.1% (95% CI: 65.4 to 80.6) for three doses administered within less than 120 days; 75.2% (95% CI, 68.1 to 80.7) for three doses administered within more than 120 days; 83.1% (95% CI, 77.3 to 87.4) for four or five doses of the original vaccine; and 88.0% (95% CI, 78.2 to 93.3) for four or five doses of the updated bivalent vaccine (Figure 2). Looking at the age subgroups, the protection conferred by three doses administered within more than 120 days remained high for patients aged 50–79 years, while it dropped to 58.8% (95% CI, 41.2 to 71.1) for patients 80 years and older. The effectiveness of four or five doses of the updated bivalent vaccine was remarkable among both patients who were 50–79 years old and 80 years or older (Figure 2). The estimates of vaccine effectiveness remained virtually unchanged in the sensitivity analyses (Table S3).

### 3.3. Vaccine Effectiveness on ICU Admissions

**Figure 3:**
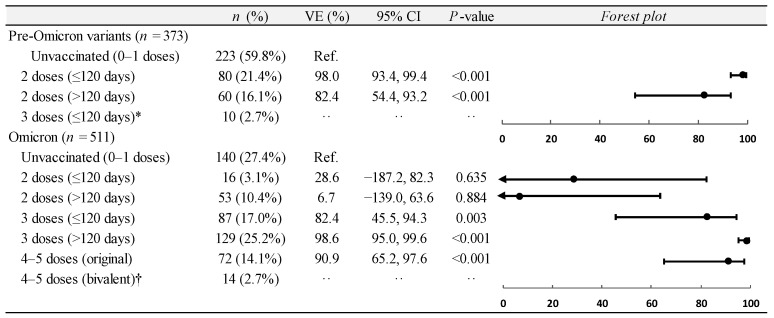
Estimates of average vaccine effectiveness (VE) against COVID-19-associated ICU admission among adults aged ≥50 years during predominance periods of SARS-CoV-2 pre-Omicron and Omicron variants (1 January to 15 December 2021 vs. 16 December 2021 to 31 January 2023). * Excluded from regression analysis due to complete separation (10 out of 10 test-negative controls). † Excluded from regression analysis due to complete separation (14 out of 14 test-negative controls). COVID-19, coronavirus disease 2019; SARS-CoV-2, severe acute respiratory syndrome coronavirus 2; ICU, intensive care unit; CI, confidence interval.

Regarding COVID-19-associated ICU admissions, during the pre-Omicron variants’ predominance period, the vaccine effectiveness of two doses administered within less than 120 days was 98.0% (95% CI, 93.4 to 99.4) (Figure 3). The study sample was too little to assess the effectiveness of three doses administered within less than 120 days, but among the 10 hospitalizations of triple-vaccinated individuals, no one was positive for SARS-CoV-2.

During the Omicron variant’s predominance period, the effectiveness against COVID-19-associated ICU admissions was 28.6% (95% CI, −187.2 to 82.3) for two doses administered within less than 120 days; 6.7% (95% CI, −139.0 to 63.6) for two doses administered within more than 120 days; 82.4% (95% CI, 45.5 to 94.3) for three doses administered within less than 120 days; 98.6% (95% CI, 95.0 to 99.6) for three doses administered within more than 120 days; and 90.9% (95% CI, 65.2 to 97.6) for four or five doses of the original vaccine (Figure 3). Again, the study sample was too small to assess the effectiveness of four or five doses of the updated bivalent vaccine, but among the 14 hospitalizations of four- or five-dose-vaccinated subjects with the bivalent vaccines, nobody was positive for SARS-CoV-2. The estimates of vaccine effectiveness remained virtually unchanged in the sensitivity analyses (Table S4).

## 4. Discussion

We conducted this study on a sample of over 5700 hospitalizations of adults aged ≥50 years with COVID-19-like illness. Our estimates showed a high effectiveness of the vaccines in preventing COVID-19-related hospitalizations and ICU admissions.

Our results confirm the most recently published data. First, the protection conferred by four and five doses of vaccine against hospitalizations and ICU admissions was high, especially when using the updated bivalent vaccines. The effectiveness of four or five doses of vaccine was always higher than 75% and higher than 80% when bivalent vaccines were used, confirming other studies’ results [8,13,29,30,31,32,33,34]. It must be underlined, however, that in our study, the difference in effectiveness between the original vaccine and the bivalent formulation is not that high. It cannot be ruled out that this small difference is due to the fact that only just over one hundred people in this study sample received the updated bivalent vaccines.

Among elderly people (≥80 years), the vaccine efficacy was high despite this age group generally presenting a reduced response to vaccines due to progressive “immunosenescence” [35,36]. Influenza vaccines, for example, have a poor efficacy in this age group, with an effectiveness of 17–53% versus 70–90% in young adults [37,38]. Our finding is reassuring, since older people are those who are at greater risk of developing severe COVID-19 [39,40,41]. Moreover, our results highlight the importance of receiving the updated booster doses in this age group.

Our study confirms the lower effectiveness of COVID-19 vaccines against the Omicron variant compared to the pre-Omicron variants [42]. According to our data, during the predominance of the Omicron variant, two doses of vaccine were, indeed, insufficient for conferring immunity, and the protection conferred by the third dose, although still substantial, was lower than that found during the predominance of the pre-Omicron variants. Interestingly, the fourth and fifth vaccine doses conferred a high level of protection, especially using bivalent vaccines, highlighting how the effectiveness of the booster doses against severe COVID-19 remained substantial, which is consistent with other studies [13,43].

The present study should be evaluated in light of several strengths. First, the sample was large and representative of the population hospitalized within the network of hospitals throughout the Turin city area. Second, we took the eligibility period for vaccination according to the specific calendar of the different age groups into account. Third, every patient was tested for SARS-CoV-2 infection when admitted to the hospital, with a 100% test coverage. Finally, the data on vaccinations and results of SARS-CoV-2 tests were complete and reliable due to registration in the Regional and National Health Service registries. Besides these strengths, this study is also subject to a number of limitations. It was conducted at a local level. Although we adjusted for demographic and health characteristics (sex, age and CCI), unmeasured residual confounding (due to socio-economic status, educational status, professional occupation, lifestyles and risk behaviors) may have biased our estimates, limiting the generalizability of the results. The recruitment of cases and controls within hospitals could limit the generalizability of results to the overall population, due to a different propensity to seek healthcare. The evaluation of the effectiveness of the fourth and fifth doses of bivalent vaccines was underpowered due to the limited sample size (3% of the total sample), as was the evaluation of the effectiveness of the two doses of vaccines during the Omicron variant period. Although we excluded patients who were previously positive for SARS-CoV-2 from the study sample, this probably did not avoid the inclusion of people who were positive for SARS-CoV-2 before hospitalization who did not report it to the health authorities. Furthermore, information on immunocompromised status was not available. Lastly, we could not stratify the results by vaccine type. Therefore, we are unable to ascertain the actual effectiveness of the different vaccine strategies that were eventually implemented.

## 5. Conclusions

In conclusion, our study shows that the COVID-19 vaccines used in the European Union are effective in protecting patients ≥50 years from hospitalizations and ICU admissions, including the oldest subgroup and also under Omicron predominance. These results emphasize the importance for adults ≥50 years of age to complete the vaccination cycle with booster doses, as recommended by health authorities around the world.

## Figures and Tables

**Table 1:** Characteristics of SARS-CoV-2-positive vs. SARS-CoV-2-negative patients hospitalized in the Local Health Unit of Turin, Italy, during the predominance period of the SARS-CoV-2 pre-Omicron variants (1 January to 15 December 2021).

	All	Positives	Negatives	cOR (95% CI)	*p*-Value
	(*n* = 2083)	(*n* = 539)	(*n* = 1544)		
Sex					
Male	1126 (54.1%)	277 (51.4%)	849 (55.0%)	Ref.	
Female	957 (45.9%)	262 (48.6%)	695 (45.0%)	1.155 (0.949, 1.406)	0.149
Age (y), mean ± SD	78.4 ± 10.6	77.7 ± 11.0	78.7 ± 10.5	0.992 (0.983, 1.001)	0.082
Age group (y)					
50–79	937 (45.0%)	237 (44.0%)	700 (45.3%)	Ref.	
≥80	1146 (55.0%)	302 (56.0%)	844 (54.7%)	1.057 (0.868, 1.287)	0.583
COVID-19 vaccination status					
Unvaccinated (0–1 doses)	967 (46.4%)	454 (84.2%)	513 (33.2%)	Ref.	
2 doses (≤120 days)	613 (29.4%)	30 (5.6%)	583 (37.8%)	0.058 (0.039, 0.086)	<0.001
2 doses (>120 days)	383 (18.4%)	52 (9.6%)	331 (21.4%)	0.178 (0.129, 0.244)	<0.001
3 doses (≤120 days)	120 (5.8%)	3 (0.6%)	117 (7.6%)	0.029 (0.009, 0.092)	<0.001
Charlson Comorbidity Index					
1–2	93 (4.5%)	40 (7.4%)	53 (3.4%)	Ref.	
3–4	708 (34.0%)	241 (44.7%)	467 (30.2%)	0.684 (0.441, 1.061)	0.090
≥5	1282 (61.5%)	258 (47.9%)	1024 (66.3%)	0.334 (0.217, 0.515)	<0.001
ICU admission					
No	1710 (82.1%)	357 (66.2%)	1353 (87.6%)	Ref.	
Yes	373 (17.9%)	182 (33.8%)	191 (12.4%)	3.611 (2.857, 4.564)	<0.001

SARS-CoV-2, severe acute respiratory syndrome coronavirus 2; cOR, crude odds ratio; CI, confidence interval; SD, standard deviation; COVID-19, coronavirus disease 2019; ICU, intensive care unit.

**Table 2:** Characteristics of SARS-CoV-2-positive vs. SARS-CoV-2-negative patients hospitalized in the Local Health Unit of Turin, Italy, during the predominance period of the SARS-CoV-2 Omicron variants (16 December 2021 to 31 January 2023).

	All	Positives	Negatives	cOR (95% CI)	*p*-Value
	(*n* = 3685)	(*n* = 1313)	(*n* = 2372)		
Sex					
Male	1904 (51.7%)	703 (53.5%)	1201 (50.6%)	Ref.	
Female	1781 (48.3%)	610 (46.5%)	1171 (49.4%)	0.890 (0.778, 1.019)	0.091
Age (y), mean ± SD	78.2 ± 11.0	78.6 ± 11.1	78.0 ± 11.0	1.006 (0.999, 1.012)	0.081
Age group (y)					
50–79	1730 (46.9%)	593 (45.2%)	1137 (47.9%)	Ref.	
≥80	1955 (53.1%)	720 (54.8%)	1235 (52.1%)	1.118 (0.976, 1.280)	0.107
COVID-19 vaccination status					
Unvaccinated (0–1 doses)	642 (17.4%)	388 (29.6%)	254 (10.7%)	Ref.	
2 doses (≤120 days)	95 (2.6%)	44 (3.4%)	51 (2.2%)	0.565 (0.366, 0.871)	0.010
2 doses (>120 days)	337 (9.1%)	174 (13.3%)	163 (6.9%)	0.699 (0.536, 0.912)	0.008
3 doses (≤120 days)	666 (18.1%)	180 (13.7%)	486 (20.5%)	0.242 (0.192, 0.306)	<0.001
3 doses (>120 days)	1093 (29.7%)	321 (24.4%)	772 (32.5%)	0.272 (0.222, 0.334)	<0.001
4–5 doses (original)	741 (20.1%)	188 (14.3%)	553 (23.3%)	0.223 (0.177, 0.280)	<0.001
4–5 doses (bivalent)	111 (3.0%)	18 (1.4%)	93 (3.9%)	0.127 (0.075, 0.215)	<0.001
Charlson Comorbidity Index					
1–2	184 (5.0%)	76 (5.8%)	108 (4.6%)	Ref.	
3–4	1399 (38.0%)	638 (48.6%)	761 (32.1%)	1.191 (0.872, 1.627)	0.271
≥5	2102 (57.0%)	599 (45.6%)	1503 (63.4%)	0.566 (0.416, 0.771)	<0.001
ICU admission					
No	3174 (86.1%)	1113 (84.8%)	2061 (86.9%)	Ref.	
Yes	511 (13.9%)	200 (15.2%)	311 (13.1%)	1.191 (0.983, 1.443)	0.075

SARS-CoV-2, severe acute respiratory syndrome coronavirus 2; cOR, crude odds ratio; CI, confidence interval; SD, standard deviation; COVID-19, coronavirus disease 2019; ICU, intensive care unit.

## Data Availability

The data supporting this article were provided by the Turin Local Health Authority. Data will be shared on request to the corresponding author with the permission of the Turin Local Health Authority.

## Supplementary Materials

The following supporting information can be downloaded at https://www.mdpi.com/article/10.3390/vaccines12111245/s1: Table S1: Distribution of COVID-19 Vaccines Administered to the Study Sample, Overall and by Time Period; Table S2: Estimates of average vaccine effectiveness (VE) against COVID-19-associated hospitalization among adults aged ≥50 years during the predominance period of SARS-CoV-2 pre-Omicron variants (January 1 to December 15, 2021), overall and by age group; Table S3: Estimates of average vaccine effectiveness (VE) against COVID-19-associated hospitalization among adults aged ≥50 years during the predominance period of SARS-CoV-2 Omicron variants (December 16, 2021 to January 31, 2023), overall and by age group; Table S4: Estimates of average vaccine effectiveness (VE) against COVID-19-associated ICU admission among adults aged ≥50 years during the predominance periods of SARS-CoV-2 pre-Omicron and Omicron variants (1 January to 15 December 2021 vs. 16 December 2021 to 31 January 2023).
