# Supplementary material for: The Effectiveness of COVID-19 Vaccines During the Pre-Omicron and Omicron Periods: A Retrospective Test-Negative Case–Control Study
Source: Vaccines (Basel). 2024 Oct 31;12(11):1245. doi: 10.3390/vaccines12111245 (PMC11598972; doi:10.3390/vaccines12111245)
Supplement: Supplementary file 1 [file vaccines-12-01245-s001.zip › vaccines-3279091-supplementary.pdf]

SUPPLEMENTS

Table S1. Distribution of COVID-19 Vaccines Administered to the Study Sample, Overall and by Time Period.

First dose	Second dose	Third dose	Fourth dose	Fifth dose	All (n = 5768)	Pre-Omicron (n = 2083)	Omicron (n = 3685)
Class 1: Unvaccinated (0–1 Dose)					1609 (27,9%)	967 (46,4%)	642 (17,4%)
-	-	-	-	-	1291	709	582
AZ	-	-	-	-	50	50	0
MD	-	-	-	-	41	17	24
PF	-	-	-	-	227	191	36
Class 2: Two Doses (≤120 Days)					708 (12,3%)	613 (29,4%)	95 (2,6%)
AZ	AZ	-	-	-	71	56	15
AZ	MD	-	-	-	1	1	0
AZ	PF	-	-	-	3	1	2
JJ*	-	-	-	-	76	73	3
MD	MD	-	-	-	42	25	17
PF	PF	-	-	-	515	457	58
Class 3: Two Doses (>120 Days)					720 (12,5%)	383 (18,4%)	337 (9,1%)
AZ	AZ	-	-	-	48	17	31
AZ	MD	-	-	-	2	0	2
AZ	PF	-	-	-	3	1	2
JJ*	-	-	-	-	96	43	53
MD	MD	-	-	-	52	12	40
MD	PF	-	-	-	2	0	2
PF	MD	-	-	-	1	0	1
PF	PF	-	-	-	516	310	206
Class 4: Three Doses (≤120 Days)					786 (13,6%)	120 (5,8%)	666 (18,1%)
AZ	AZ	MD	-	-	35	0	35
AZ	AZ	PF	-	-	38	0	38
AZ	MD	MD	-	-	1	0	1
AZ	PF	MD	-	-	1	0	1
AZ	PF	PF	-	-	4	0	4
JJ*	-	MD	-	-	15	1	14
JJ*	-	PF	-	-	57	1	56
MD	MD	MD	-	-	29	1	28
MD	MD	PF	-	-	13	1	12
MD	MD	PFBA1	-	-	1	0	1
PF	MD	PF	-	-	1	0	1
PF	PF	MD	-	-	213	19	194
PF	PF	PF	-	-	377	97	280
PF	PF	PFBA1	-	-	1	0	1
Class 5: Three Doses (>120 Days)					1093 (18,9%)	0 (0,0%)	1093 (29,7%)
AZ	AZ	MD	-	-	68	0	68
AZ	AZ	PF	-	-	59	0	59
AZ	MD	MD	-	-	1	0	1
AZ	PF	MD	-	-	4	0	4
AZ	PF	PF	-	-	4	0	4
JJ*	-	MD	-	-	11	0	11
JJ*	-	PF	-	-	66	0	66
MD	MD	MD	-	-	42	0	42

MD	MD	PF	-	-	27	0	27
PF	AZ	PF	-	-	1	0	1
PF	MD	MD	-	-	1	0	1
PF	PF	MD	-	-	326	0	326
PF	PF	PF	-	-	483	0	483
Class 6: Four of Five Doses (Original)					741 (12,8%)	0 (0,0%)	741 (20,1%)
AZ	AZ	MD	MD	-	5	0	5
AZ	AZ	MD	PF	-	9	0	9
AZ	AZ	PF	PF	-	9	0	9
JJ*	-	MD	MD	-	1	0	1
JJ*	-	MD	PF	-	7	0	7
JJ*	-	PF	PF	-	45	0	45
MD	MD	MD	MD	-	7	0	7
MD	MD	MD	PF	-	8	0	8
MD	MD	PF	MD	-	5	0	5
MD	MD	PF	PF	-	7	0	7
PF	PF	MD	MD	-	65	0	65
PF	PF	MD	PF	-	151	0	151
PF	PF	PF	MD	-	168	0	168
PF	PF	PF	PF	-	249	0	249
PF	PF	PF	MD	PF	5	0	5
Class 7: Four of Five Doses (Bivalent)					111 (1,9%)	0	111 (3,0%)
AZ	AZ	MD	PFBA1	-	7	0	7
AZ	AZ	MD	PFBA45	-	1	0	1
AZ	AZ	PF	PFBA1	-	8	0	8
AZ	AZ	PF	PFBA45	-	1	0	1
AZ	PF	PF	PFBA1	-	1	0	1
JJ*	-	MD	PFBA1	-	1	0	1
JJ*	-	MD	PFBA45	-	1	0	1
JJ*	-	PF	PFBA1	-	3	0	3
MD	MD	MD	PFBA1	-	4	0	4
MD	MD	PF	PFBA1	-	1	0	1
PF	PF	MD	PFBA1	-	13	0	13
PF	PF	MD	PFBA45	-	1	0	1
PF	PF	PF	PFBA1	-	12	0	12
PF	PF	PF	PFBA45	-	1	0	1
PF	PF	MD	MD	PFBA1	4	0	4
PF	PF	MD	MD	PFBA45	9	0	9
PF	PF	MD	PF	PFBA1	6	0	6
PF	PF	MD	PF	PFBA45	3	0	3
PF	PF	PF	MD	PFBA1	8	0	8
PF	PF	PF	MD	PFBA45	10	0	10
PF	PF	PF	PF	PFBA1	8	0	8
PF	PF	PF	PF	PFBA45	8	0	8

Abbreviations: AZ, ChAdOx1-S; JJ, Ad26.COV2.S; MD, Original mRNA-1273; PF, Original BNT162b2; PFBA1, Comirnaty Original/Omicron BA.1; PFBA45 Comirnaty Original/Omicron BA.4-5.

*Since Ad26.COV2.S is a monodose vaccine, one dose of Ad26.COV2.S is considered as full course vaccination (as if the patient had had two doses of another vaccine).

Table S2. Estimates of average vaccine effectiveness (VE) against COVID-19-associated hospitalization among adults aged ≥50 years during the predominance period of SARS-CoV-2 pre-Omicron variants (January 1 to December 15, 2021), overall and by age group.

	n (%)	VE (%)	95% CI	*P*-value
All (*n* = 2083)				
Unvaccinated (0–1 doses)	967 (46.4)	Ref.		
2 doses (<120 days)	613 (29.4)	94.2	91.1, 96.2	<0.001
2 doses (>120 days)	383 (18.4)	81.1	73.6, 86.5	<0.001
3 doses (<120 days)	120 (5.8)	97.6	91.2, 99.3	<0.001
50–79 years (*n* = 937)				
Unvaccinated (0–1 doses)	455 (48.6)	Ref.		
2 doses (<120 days)	301 (32.1)	93.2	87.6, 96.3	<0.001
2 doses (>120 days)	147 (15.7)	77.8	62.9, 86.7	<0.001
3 doses (<120 days)	34 (3.6)	96.2	68.0, 99.5	0.003
≥80 years (*n* = 1146)				
Unvaccinated (0–1 doses)	512 (44.7)	Ref.		
2 doses (<120 days)	312 (27.2)	95.4	91.3, 97.5	<0.001
2 doses (>120 days)	236 (20.6)	84.3	75.6, 89.9	<0.001
3 doses (<120 days)	86 (7.5)	98.3	90.6, 99.7	<0.001

Notes: Propensity-for-vaccination scores were estimated using all study observations, not only test-negative controls.
COVID-19, coronavirus disease 2019; *SARS-CoV-2*, severe acute respiratory syndrome coronavirus 2; *CI*, confidence interval.

Table S3. Estimates of average vaccine effectiveness (VE) against COVID-19-associated hospitalization among adults aged ≥50 years during the predominance period of SARS-CoV-2 Omicron variants (December 16, 2021 to January 31, 2023), overall and by age group.

	n (%)	VE (%)	95% CI	*P*-value
All (*n* = 3685)				
Unvaccinated (0–1 doses)	642 (17.4%)	Ref.		
2 doses (<120 days)	95 (2.6%)	35.4	−3.8, 59.8	0.071
2 doses (>120 days)	337 (9.1%)	26.5	1.3, 45.3	0.041
3 doses (<120 days)	666 (18.1%)	73.6	65.6, 79.7	<0.001
3 doses (>120 days)	1093 (29.7%)	69.3	61.3, 75.7	<0.001
4–5 doses (original)	741 (20.1%)	78.3	71.7, 83.4	<0.001
4–5 doses (bivalent)	111 (3.0%)	86.6	76.5, 92.4	<0.001
50–79 years (*n* = 1730)				
Unvaccinated (0–1 doses)	346 (20.0%)	Ref.		
2 doses (<120 days)	65 (3.8%)	47.7	5.7, 71.0	0.031
2 doses (>120 days)	174 (10.1%)	37.1	4.5, 58.6	0.030
3 doses (<120 days)	321 (18.6%)	80.7	71.6, 86.9	<0.001
3 doses (>120 days)	549 (31.7%)	80.1	72.0, 85.8	<0.001
4–5 doses (original)	200 (11.6%)	84.9	75.9, 90.6	<0.001
4–5 doses (bivalent)	75 (4.3%)	91.1	81.1, 95.8	<0.001
≥80 years (*n* = 1955)				
Unvaccinated (0–1 doses)	296 (15.1%)	Ref.		
2 doses (<120 days)	30 (1.5%)	28.7	−57.2, 67.7	0.402
2 doses (>120 days)	163 (8.3%)	16.7	−26.3, 45.0	0.390
3 doses (<120 days)	345 (17.6%)	65.8	50.7, 76.3	<0.001
3 doses (>120 days)	544 (27.8%)	56.6	39.9, 68.7	<0.001
4–5 doses (original)	541 (27.7%)	70.8	59.2, 79.1	<0.001
4–5 doses (bivalent)	36 (1.8%)	82.0	56.5, 92.5	<0.001

Notes: Propensity-for-vaccination scores were estimated using all study observations, not only test-negative controls.
COVID-19, coronavirus disease 2019; *SARS-CoV-2*, severe acute respiratory syndrome coronavirus 2; *CI*, confidence interval.

Table S4. Estimates of average vaccine effectiveness (VE) against COVID-19-associated ICU admission among adults aged ≥50 years during the predominance periods of SARS-CoV-2 pre-Omicron and Omicron variants (January 1 to December 15, 2021 vs. December 16, 2021 to January 31, 2023).

	n (%)	VE (%)	95% CI	*P*-value
Pre-Omicron variants (*n* = 373)				
Unvaccinated (0–1 doses)	223 (59.8%)	Ref.		
2 doses (<120 days)	80 (21.4%)	96.0	90.4, 98.4	<0.001
2 doses (>120 days)	60 (16.1%)	86.1	71.9, 93.1	<0.001
3 doses (<120 days)*	10 (2.7%)	‥	‥	‥
Omicron (*n* = 511)				
Unvaccinated (0–1 doses)	140 (27.4%)	Ref.		
2 doses (<120 days)	16 (3.1%)	72.1	6.7, 91.7	0.038
2 doses (>120 days)	53 (10.4%)	40.4	−20.5, 70.5	0.149
3 doses (<120 days)	87 (17.0%)	76.2	54.3, 87.6	<0.001
3 doses (>120 days)	129 (25.2%)	85.8	74.0, 92.2	<0.001
4–5 doses (original)	72 (14.1%)	89.7	75.4, 95.7	<0.001
4–5 doses (bivalent)†	14 (2.7%)	‥	‥	‥

Notes: Propensity-for-vaccination scores were estimated using all study observations, not only test-negative controls.
*Excluded from regression analysis due to complete separation (10 out of 10 test-negative controls).
†Excluded from regression analysis due to complete separation (14 out of 14 test-negative controls).
COVID-19, coronavirus disease 2019; *SARS-CoV-2*, severe acute respiratory syndrome coronavirus 2; *ICU*, intensive care unit; *CI*, confidence interval.
